# Comparing the performance of the EQ-5D and SF-6D when measuring the benefits of alleviating knee pain

**DOI:** 10.1186/1478-7547-7-12

**Published:** 2009-07-17

**Authors:** Garry R Barton, Tracey H Sach, Anthony J Avery, Michael Doherty, Claire Jenkinson, Kenneth R Muir

**Affiliations:** 1 Health Economics Group, School of Medicine, Health Policy and Practice, University of East Anglia, Norwich, UK; 2 School of Chemical Sciences and Pharmacy, University of East Anglia, Norwich, UK; 3 School of Community Health Sciences, University of Nottingham, Nottingham, UK; 4 Academic Rheumatology, University of Nottingham, Nottingham UK

## Abstract

**Objective:**

To assess the practicality, validity and responsiveness of using each of two utility measures (the EQ-5D and SF-6D) to measure the benefits of alleviating knee pain.

**Methods:**

Participants in a randomised controlled trial, which was designed to compare four different interventions for people with self-reported knee pain, were asked to complete the EQ-5D, SF-6D, and Western Ontario and McMaster Universities Osteoarthritis Index (WOMAC) at both pre- and post-intervention. For both utility measures, we assessed their practicality (completion rate), construct validity (ability to discriminate between baseline WOMAC severity levels), and responsiveness (ability to discriminate between three groups: those whose total WOMAC score, i) did not improve, ii) improved by <20%, and iii) improved by ≥20%).

**Results:**

The EQ-5D was completed by 97.7% of the 389 participants, compared to 93.3% for the SF-6D. Both the EQ-5D and SF-6D were able to discriminate between participants with different levels of WOMAC severity (p < 0.001). The mean EQ-5D change was -0.036 for group i), 0.091 for group ii), and 0.127 for group iii), compared to 0.021, 0.023 and 0.053 on the SF-6D. These change scores were significantly different according to the EQ-5D (p < 0.001), but not the SF-6D.

**Conclusion:**

The EQ-5D and SF-6D had largely comparable practicality and construct validity. However, in contrast to the EQ-5D, the SF-6D could not discriminate between those who improved post-intervention, and those who did not. This suggests that it is more appropriate to use the EQ-5D in future cost-effectiveness analyses of interventions which are designed to alleviate knee pain.

**Trial registration:**

Current Controlled Trials ISRCTN93206785

## Background

In the UK it has been estimated that nearly 50% of those aged >50 years experience knee pain each year, and that 33% of these consequently consult their general practitioner [1]. Economic evaluations have accordingly been undertaken to assess whether interventions which alleviate knee pain represent a cost-effective use of scarce health care resources [2]. Within such studies outcomes are often measured on a utility scale, where 0 is equivalent to death and 1 is equal to full health, in order to enable the benefits of different interventions to be compared on a common scale [3,4] There are however a number of different utility measures that can be used within such evaluations, including the EQ-5D [5], health utilities index [6], and SF-6D [7], all of which aim to measure utility on the same scale. Moreover, as each of these measures are based on different health descriptions [8], and different valuation methods [9], there is increasing evidence that they produce different results [10]. Fitzpatrick et al. [11] outlined a number of criteria (appropriateness, reliability, validity, responsiveness, precision, interpretability, acceptability, feasibility) on which evidence should be provided in order to select an appropriate outcome measure, and very few papers have assessed utility measures with regard to such criteria [12]. Thus, within this paper we seek to compare the performance of the EQ-5D and SF-6D with regard to the criteria of practicality, validity and responsiveness. The results of this study are particularly important as both of these measures have recently been used in a randomised controlled trial which compared four interventions for people with knee pain (diet and strengthening exercise advice, dietary advice, strengthening exercise advice, and leaflet provision) and we wish to select the preferred outcome measure for the cost-effectiveness analysis of this study in a systematic and transparent way.

The importance of such an analysis is further highlighted by two potentially opposed views. Firstly, the National Institute of Health and Clinical Excellence (NICE) has recently stated that the EQ-5D is the preferred measure of utility to be used in economic evaluations [13]. However, it did acknowledge that an alternative measure could be used if empirical evidence can be used to show that it is not suitable for a particular patient group, where relevant properties include practicality, validity and responsiveness [13]. Secondly, it has been argued that it might not be appropriate to use the EQ-5D in a rheumatology clinic group [14]. The basis for this latter view was that the EQ-5D was less responsive to change, than other measures in the study [14]. Moreover, the possible inappropriateness of the EQ-5D is also highlighted by two recent studies [2,15], in similar population groups, which found that the interventions in question were associated with an improvement according to a clinical measure, but a reduced post-intervention score according to the EQ-5D. Thus, here we seek to compare the performance of the EQ-5D and SF-6D in a group of patients with self-reported knee pain.

## Methods

### Participants

All participants were taking part in the Lifestyle Interventions for Knee Pain (LIKP) study, which was designed to compare the effectiveness and cost-effectiveness of four different interventions (receipt of a leaflet, dietary advice, guidance on knee strengthening exercises, or dietary advice and guidance on knee strengthening exercises). Ethical approval for this study was granted by the UK Nottingham Research Ethics Committee. In order to recruit people into the LIKP study all registered patients in five Nottingham general practices who were aged ≥45 years, and deemed (by their general practitioner) to be well enough to complete a questionnaire, were sent an ascertainment questionnaire, and a local media campaign was also conducted. Responding individuals were recruited into the LIKP study if they reported that they had had knee pain on most days of the last month, were aged ≥45 years, had a body mass index (BMI) >28.0 kg/m^2^, and gave consent to be randomised to one of the four interventions.

#### Outcome Measures

Participants in the LIKP study were asked to complete, amongst other things, three outcome questionnaire measures at both pre- and (6 months) post-intervention – the WOMAC (Western Ontario and McMaster Universities Osteoarthritis Index), EQ-5D and SF-36 (the latter was used to calculate the SF-6D score). The WOMAC was chosen as primary outcome measure within the LIKP study as the pain subscale of the WOMAC was considered to be the best way of capturing knee pain severity.

The WOMAC measures the amount of pain (5 questions), stiffness (2 questions), and difficulty in physical functioning (17 questions), where the response options are none (0), mild (1), moderate (2), severe (3) or extreme (4) [16]. Scores can thereby range between 0 and 20 on the pain scale, 0 and 8 on the stiffness scale, 0 and 68 on the functioning scale, and 0 to 96 on the total WOMAC (WOMAC_96_) scale, where higher scores denote a worse response [17]. Previous evidence of the adequate performance of the WOMAC has been shown for construct validity [18] and responsiveness [19,20].

The EQ-5D has five questions, where the respondent is asked to report the level of problems they have (no problems, some/moderate problems, and severe/extreme problems) with regard to mobility, self-care, usual activities, pain, and anxiety/depression [5]. Responses to these five dimensions are converted into one of 243 different EQ-5D health state descriptions, which range between no problems on all five dimensions (11111) and severe/extreme problems on all five dimensions (33333). A utility score was assigned to each health state using the York A1 tariff [21], which was based on the preferences elicited from a survey of 3395 UK residents – EQ-5D scores range between -0.594 and 1 (full health).

In a similar way, responses to eleven of the questions on the SF-36 [22] were used to estimate a score on the SF-6D [7]. The SF-6D is composed of six dimensions (physical functioning, role limitations, social functioning, pain, mental health and vitality) which have between four and six levels. We used the consistent [23] version of the SF-6D algorithm [8] to estimate utility scores for each of 18,000 potential health states – SF-6D scores range between 0.296 and 1.00.

### Comparing the EQ-5D and SF-6D – performance criteria

#### Choice of Analysis

There are many approaches to assessing validity and, as Fitzpatrick et al. [11] point out, these criteria are not uniformly described. Indeed, Streiner & Norman [24] suggest that the myriad of terms that are used to describe such approaches means that one of the most difficult aspects of validity testing is the terminology. In the light of this, we attempt to provide clear definitions of the type of validity that we are testing for in order to avoid the possibility of misinterpretation. References to previous studies which have used similar techniques are also provided. Finally, rather than assessing the predictive ability of certain variables [25], it should be noted that we focus solely on the relationship between the WOMAC and the utility measures of the EQ-5D and SF-6D as, as far as we are aware, such relationships have not been previously investigated. This is in contrast to a number of previous studies e.g. [26,27] which have looked at the effect that different socio-demographic characteristics and clinical conditions have on measures of utility.

#### Practicality

Practicality was assessed in terms of completion rates, where the SF-36 appeared before the EQ-5D in the ascertainment questionnaire. We assessed whether sufficient information was provided in order to calculate a utility score for the EQ-5D and SF-6D, as outlined by Gerard et al. [28].

#### Validity

Validity was assessed in terms of both construct and convergent validity. Construct validity relates to whether a measure can discriminate between two patient groups, one which has a certain trait, and the other which does not [24]. This has also been referred to as known groups validity [29,30], based on the principle that certain specified groups of patients may be expected to score differently from one another. We assessed whether the EQ-5D and SF-6D could discriminate between participants with different levels of (pre-intervention) severity on the WOMAC. In accordance with the WOMAC response options (none (0), mild (1), moderate (2), severe (3) or extreme (4)), four severity levels were created on the overall WOMAC_96 _score – i) none to mild (total score of 0 to ≤24 on the WOMAC_96 _scale), ii) >mild to moderate (total score of >24 to ≤48 on the WOMAC_96 _scale), iii) >moderate to severe (total score of >49 to ≤72 on the WOMAC_96 _scale), and iv) >severe to extreme (total score of >72 to ≤96 on the WOMAC_96 _scale). Similar severity levels were also created for each of the three sub-scales: i) none to mild was denoted by total scores of 0 to ≤5 (pain), 0 to ≤2 (stiffness), and 0 to ≤17 (functioning), ii) >mild to moderate was denoted by total scores of >5 to ≤10 (pain), >2 to ≤4 (stiffness), and >17 to ≤34, iii) >moderate to severe was denoted by total scores of >10 to ≤15 (pain), >4 to ≤6 (stiffness), and >34 to ≤51 (functioning), and iv) >severe to extreme was denoted by total scores of >15 to ≤20 (pain), >6 to ≤8 (stiffness), and >51 to ≤68 (functioning). On the overall WOMAC_96 _score, and each of the three WOMAC subscales, in order to assess whether there were significant (p < 0.05) differences between the utility scores of participants in each of these four severity levels a one-way analysis of variance (ANOVA) was conducted. This analysis is akin to that conducted previously [30,31].

Convergent validity is determined by how closely a measure is related to other measures of the same construct [24]. Thus, in line with previous studies [30,32,33], we assessed whether (pre-intervention) scores on the EQ-5D and SF-6D were significantly correlated with the WOMAC_96 _score according to the Spearman rank test.

#### Responsiveness

Responsiveness, which is different to sensitivity [29], is determined by the ability an instrument has to detect a meaningful or clinically important change [34], where one seeks to discriminate between those who change a lot and those who change a little [24]. Previously, a 20% improvement on each of the three subscales of the WOMAC has been deemed to equate to the minimum clinically important difference [35-37]. Thus, we sought to assess whether the EQ-5D and SF-6D could discriminate between three groups: i) those who did not improve according to the WOMAC (≤0% change post-intervention), ii) those who improved by <20% (>0% to <20% change post-intervention), and iii) those who improved by ≥20% (≥20% change post-intervention) – the change was estimated by subtracting the pre-intervention score from the post-intervention score, and those who had a worse WOMAC score post-intervention were included in the first group. The mean change scores for the EQ-5D and SF-6D were calculated for each of these three post-intervention groups, for both the overall WOMAC_96 _scale and each of the three WOMAC subscales, and the paired t-tests was conducted to assess whether there was a significant change in the mean utility score. For both the EQ-5D and the SF-6D a one-way ANOVA was also conducted to assess whether there was a significant difference between the mean change in utility across each of the three groups. The above analysis is in line with that undertaken previously [30].

## Results

### Participants

Questionnaires were returned by 8,044 of the 12,500 people (64.4%) who were sent an ascertainment questionnaire. Of these, 318 were eligible to take part in the LIKP study and consented to be randomised to one of the four interventions. An additional 71 participants were recruited via the media campaign. The mean age of these 389 participants was 62.0 years, 66.0% were female, and 23.4% were classified as overweight (BMI 25 to <30 kg/m^2^), 50.4% as class I obese (30 to <35 kg/m^2^), 16.9% as class II obese (35 to <40 kg/m^2^), and 9.9% as class III obese (≥40 kg/m^2^). Pre-intervention the mean score on each of the pain, stiffness and functioning dimensions of the WOMAC was 7.81 (N = 360), 3.92 (N = 360), and 27.90 (N = 359), respectively, the mean WOMAC_96 _score was 39.59 (N = 359).

### Comparing the performance of the EQ-5D and SF-6D

#### Practicality

Pre-intervention the EQ-5D was wholly completed by 378 of the 389 participants (97.2%). Four of these participants were categorised as being in full health (11111), and a total of 36 different EQ-5D health states were reported – 29 participants had health states rated as worse than death, the lowest score was -0.239 (22333), and the mean score was 0.550 (95% confidence interval 0.521 to 0.578).

Pre-intervention, SF-6D scores could be calculated for 366 of the 389 participants (94.1%). None were categorised in full health, but one person did report that they had the lowest score on all six dimensions (645655). SF-6D scores ranged from 0.296 to 0.948, 194 different health states were reported, and the mean score was 0.646 (95% confidence interval 0.631 to 0.660).

#### Validity

In terms of construct validity the results in Table 1 show that, for the 359 participants for whom the WOMAC_96 _could be calculated (pre-intervention), participants who had higher scores (increased severity) tended to have lower utility scores on both the EQ-5D and SF-6D – the mean EQ-5D (SF-6D) utility score for those with a WOMAC_96 _score between 0 and 24 was 0.722 (0.731), compared to 0.069 (0.460) for those with a WOMAC_96 _score between 73 and 96. These differences were significant according to the one-way ANOVA, and similar results were also obtained for each of the three WOMAC subscales (Table 1). With regard to convergent validity, scores on both the EQ-5D and SF-6D scores were highly correlated (p < 0.001) with scores on the WOMAC_96 _scale (r = -0.576 and r = -0.501, respectively).

**Table 1:** Construct validity: Mean EQ-5D and SF-6D utility scores for each of the four baseline severity levels.

WOMAC severity level	Pain		Stiffness		Functioning		Overall (WOMAC_96_)	
	
	EQ-5D	SF-6D	EQ-5D	SF-6D	EQ-5D	SF-6D	EQ-5D	SF-6D
none to mild	0.696 (96)	0.714 (93)	0.672 (67)	0.686 (65)	0.722 (78)	0.732 (75)	0.722 (69)	0.731 (67)
>mild to moderate	0.572 (168)	0.658 (160)	0.602 (164)	0.676 (157)	0.606 (150)	0.671 (147)	0.618 (165)	0.679 (160)
>moderate to severe	0.383 (80)	0.567 (79)	0.462 (108)	0.601 (106)	0.409 (111)	0.574 (106)	0.390 (107)	0.562 (103)
>severe to extreme	0.092‡ (5)	0.471‡ (5)	0.027‡ (10)	0.477‡ (9)	0.148‡ (9)	0.478‡ (8)	0.069‡ (7)	0.460‡ (6)

The number of participants in each group are reported in brackets (N), results of the ANOVA are also noted (‡ p < 0.001).

#### Responsiveness

Pre- and post-intervention WOMAC_96 _scores could be calculated for 324 participants. Post-intervention the WOMAC_96 _score did not improve for 33.8% of the participants, for 25.2% the WOMAC_96 _score improved by <20%, and for 40.9% the WOMAC_96 _score improved by ≥20%. The mean change in utility (post-intervention) for each of these three groups was -0.036, 0.091 and 0.127 for the EQ-5D, compared to 0.021, 0.023 and 0.053 for the SF-6D (Table 2). The one-way ANOVA showed that the differences between these three groups were significant according to the EQ-5D (p < 0.001), but not the SF-6D (p = 0.084). Similar results were also obtained across the three subscales of the WOMAC (see Table 2).

**Table 2:** Responsiveness: Mean EQ-5D and SF-6D changes scores for each of the three post-intervention groups.

	Pain		Stiffness		Functioning		Overall (WOMAC_96_)	
WOMAC change	EQ-5D	SF-6D	EQ-5D	SF-6D	EQ-5D	SF-6D	EQ-5D	SF-6D

No improvement	-0.030 (124)	0.022* (123)	0.024 (156)	0.029‡ (152)	-0.018 (114)	0.021* (111)	-0.036 (103)	0.021 (100)
Improved <20%	0.140† (48)	0.031 (45)	0.038 (17)	0.014 (15)	0.072* (71)	0.026* (67)	0.091† (80)	0.023 (78)
Improved ≥ 20%	0.119‡ (141)	0.045‡ (134)	0.110‡ (141)	0.042‡ (136)	0.132‡ (127)	0.050‡ (123)	0.127‡ (128)	0.053‡ (122)
ANOVA (F-score)	12.80‡	1.19	3.74*	0.64	9.49†	1.98	11.16‡	2.50

The number of participants in each group are reported in brackets (N), results of the paired t-tests and ANOVA are also noted (* p < 0.05, † p < 0.01, and ‡ p < 0.001).

## Discussion

When comparing the performance of the EQ-5D and SF-6D with regard to the criteria of practicality we found that the completion rate (pre-intervention) was lower for the SF-6D, even though the SF-36 appeared first in the ascertainment questionnaire. In terms of validity both the EQ-5D and SF-6D were able to discriminate between groups with different levels of severity according to the WOMAC, and were also highly correlated with the WOMAC_96 _score. However, in contrast to the EQ-5D, the SF-6D could not discriminate between participants whose condition had not improved according to the WOMAC and those who had improved by ≥20%.

### Comparisons with other studies

We are aware of only one other study which has compared the performance of the EQ-5D and SF-6D with regard to similar criteria in a similar clinical area [38]. In line with our results it was found that the EQ-5D had a higher completion rate, and that both measures were able to discriminate between groups of patients with different levels of self-reported severity, and control, of rheumatoid arthritis [38]. However, when assessing the responsiveness of the EQ-5D and SF-6D (in relation to a self-reported assessment of disease severity) they found that, on the basis of the effect size [24], the EQ-5D was more responsive in patients who (post-intervention) were classified as 'worse' but that the SF-6D was more responsive in those patients classified as 'better' [38]. For the EQ-5D, other results in similar clinical areas concur with our findings – the EQ-5D was able to discriminate between patients with different severity levels of knee osteoarthritis [14] and patients with different levels of functional class according to the Stanford Health Assessment Questionnaire [39]. EQ-5D scores have also been shown to be highly correlated with many measures from the American College of Rheumatology (ACR) disease activity set [39], and the WOMAC [40-42]. Finally, in terms of responsiveness, scores on the EQ-5D have been shown to increase for those who reported an improvement in their arthritis [39], and for rheumatoid arthritis patients who reported an improvement in pain after receiving infliximab [43].

### Limitations

Within this paper we have used the WOMAC to assess the validity and responsiveness of the EQ-5D and SF-6D. Evidence of adequate performance of the WOMAC on these criteria [18-20] justifies such an approach. However, in other patient groups, when a different condition-specific measure acts as a so called 'gold standard', the results may be different, and one should therefore be cautious about generalizing the results of this study beyond patients with knee pain. Similarly, we have only assessed the performance of the two utility measures on a limited number of criteria (the design of our study did not permit us to assess the remaining criteria outlined by Fitzpatrick et al. [11]), and thus we can not wholly conclude that the EQ-5D is superior to the SF-6D.

### Implications

Economic evaluation plays a major role in decision making [44]. Our finding that the SF-6D is less responsive to interventions designed to alleviate knee pain suggests i) that these two measures will provide different estimates of the effectiveness of different health care interventions, as has been demonstrated elsewhere [45-47], and ii) that it is more appropriate to use the EQ-5D to estimate the cost-effectiveness of interventions designed to alleviate knee pain.

The SF-6D was less responsive even though it had a greater descriptive ability (pre-intervention patients were assigned to 194 different health states on the SF-6D, compared to 36 on the EQ-5D). This is an important finding as researchers are currently investigating whether to expand the number of responses within each of the five dimensions of the EQ-5D from 3 to 5 levels [48]. One potential implication of our research is therefore that further validity checks, akin to those outlined in this paper, are needed in order to ascertain the extent to which an increased descriptive ability (which was argued to be one of the main advantages of the SF-6D [7]) results in a corresponding increase in the level of responsiveness.

## Conclusion

Though the construct and convergent validity of the EQ-5D and SF-6D were similar the EQ-5D had a higher completion rate and was more responsive. This suggests that these two measures may provide different estimates of effectiveness, and that it is more appropriate to use the EQ-5D to estimate the cost-effectiveness of alleviating knee pain.

## Competing interests

The authors declare that they have no competing interests.

## Authors' contributions

GB and TS conceived the idea for the paper, undertook the analysis and drafted the paper. CJ, AA, MD, and KM assisted in the acquisition of data, interpretation of the analysis, and commented on drafts of the manuscript. All authors read and approved the final manuscript.
